# Comparing Glucagon‐like peptide‐1 receptor agonists versus metformin in drug‐naive patients: A nationwide cohort study

**DOI:** 10.1111/1753-0407.70000

**Published:** 2024-10-04

**Authors:** Kathrine Kold Sørensen, Thomas Alexander Gerds, Lars Køber, Emil Loldrup Fosbøl, Henrik Enghusen Poulsen, Amalie Lykkemark Møller, Mikkel Porsborg Andersen, Ulrik Pedersen‐Bjergaard, Christian Torp‐Pedersen, Bochra Zareini

**Affiliations:** ^1^ Department of Cardiology Nordsjælland Hospital Hillerød Denmark; ^2^ Section of Biostatistics, Department of Public Health University of Copenhagen Copenhagen Denmark; ^3^ The Heart Centre, University Hospital of Copenhagen Denmark; ^4^ Department of Clinical Medicine University of Copenhagen Copenhagen Denmark; ^5^ Department of Endocrinology University Hospital Copenhagen Frederiksberg Denmark; ^6^ Section of Epidemiology, Department of Public Health University of Copenhagen Copenhagen Denmark; ^7^ Cancer Surveillance and Pharmacoepidemiology Danish Cancer Institute Copenhagen Denmark; ^8^ Department of Endocrinology and Nephrology, North Zealand University Hospital, Hillerød and Department of Clinical Medicine University of Copenhagen Copenhagen Denmark

## Abstract

**Background:**

Glucagon‐like peptide‐1 receptor agonists (GLP‐1 RA) are increasingly being prescribed in drug‐naive patients. We aimed to contrast add‐on therapy, adherence, and changes in biomarkers, 1 year after treatment initiation with GLP‐1 RA or metformin.

**Methods:**

Using Danish nationwide registers, we included incident GLP‐1 RA or metformin users from 2018 to 2021 with glycated hemoglobin (HbA1c) ≥ 42 mmol/mol. GLP‐1 RA initiators were matched to metformin initiators in a ratio of 1:1 to assess outcomes in prediabetes and diabetes. Main outcomes analyzed were 1‐year risk of add‐on glucose‐lowering medication and 1‐year risk of nonadherence. One‐year risks were estimated with multiple logistic regression and standardized. Multiple linear regression was used to estimate the average differences in biomarker changes.

**Results:**

In total, 1778 individuals initiating GLP‐1 RA and metformin were included. After standardizing for various factors, GLP‐1 RA compared with metformin was associated with reduced 1‐year risk of add‐on glucose‐lowering treatment in patients with prediabetes (1‐year risk ratio [RR]: 0.27, 95% confidence interval [CI]: 0.10–0.44) and diabetes (RR: 0.67, 95% CI: 0.37–0.98). GLP‐1 RA was associated with higher 1‐year risk of nonadherence among patients with prediabetes (RR: 1.60, 95% CI: 1.45–1.75), but no difference in patients with diabetes (RR: 0.88, 95% CI: 0.70–1.06). Compared to metformin, GLP‐1 RA was associated with greater HbA1c reduction (prediabetes: −2.59 mmol/mol 95% CI: −3.10 to −2.09, diabetes: −3.79 mmol/mol, 95% CI: −5.28 to −2.30).

**Conclusions:**

GLP‐1 RA was associated with a reduced risk of additional glucose‐lowering medication, achieving better glycated hemoglobin control overall. However, among patients with prediabetes, metformin was associated with better adherence.

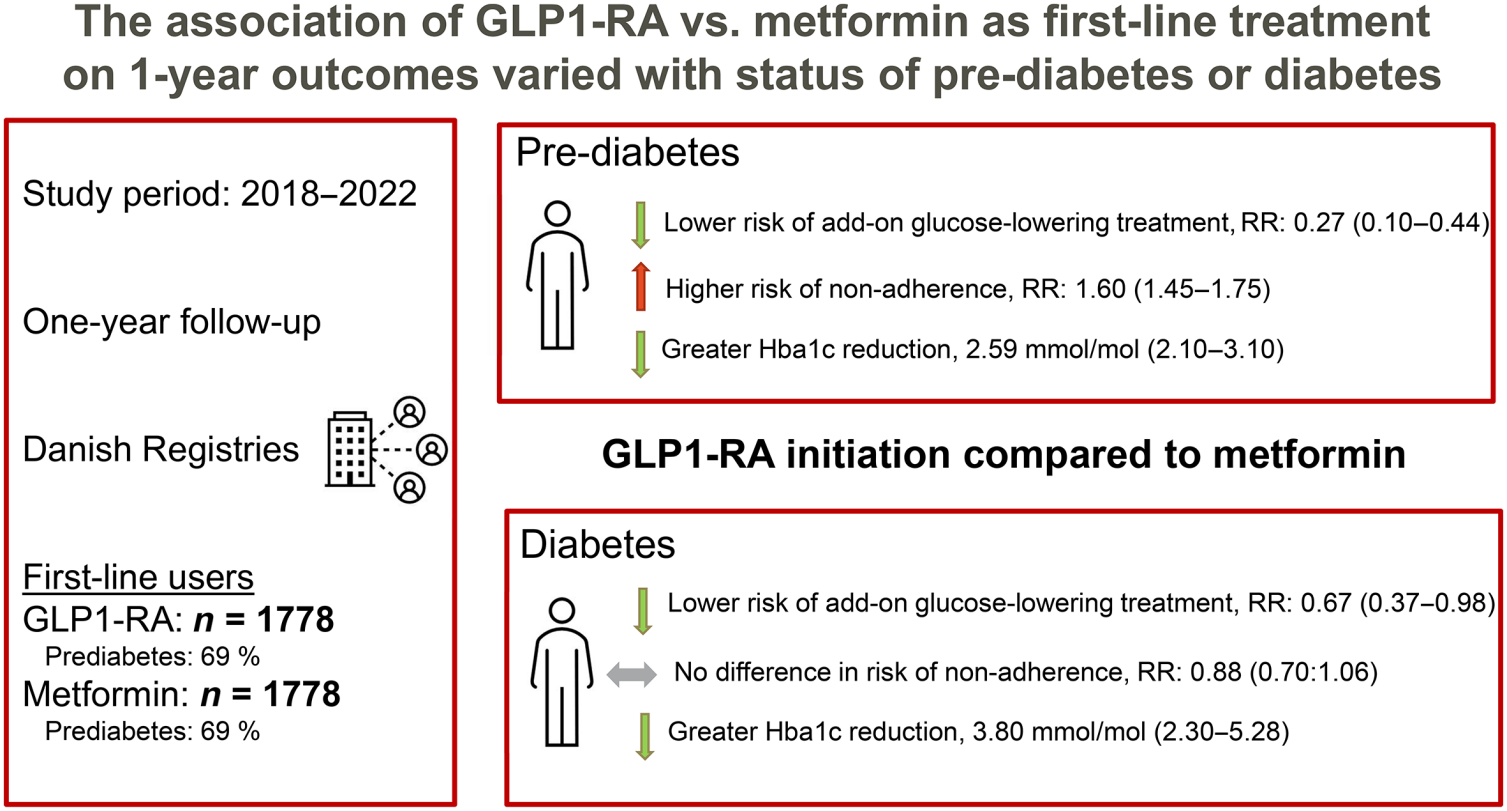

## Graphical abstract

Highlights

- In this nationwide cohort study, initiating GLP‐1 RA as the first‐line treatment was associated with improved 1‐year HbA1c levels compared with metformin.
- GLP‐1 RA initiation was also associated with a lower risk of requiring additional glucose‐lowering therapy; however, initial treatment adherence varied depending on the prediabetes or diabetes status.
- Overall, GLP‐1 RAs as the first‐line choice for 1‐year outcomes may exhibit variability regarding treatment management based on prediabetes or diabetes status, but they generally result in improved glycemic control.

## 1 INTRODUCTION

In patients with type 2 diabetes with overweight and no cardiovascular disease or moderate risk of cardiovascular disease, metformin is the recommended initial treatment (class IIa recommendation).^1^ The Food and Drug Administration (FDA) did not approve glucagon‐like peptide‐1 receptor agonists (GLP‐1 RA) (semaglutide) tablets for drug‐naive type 2 diabetes until January 2023.^2^ To our knowledge, these recommendations were not based on any head‐to‐head randomized clinical trials or postauthorization safety studies investigating the short‐term benefit on glycemic dysregulation, kidney function, and cholesterol levels.

Metformin received FDA approval in 1994 for type 2 diabetes and with its beneficial effect on glycated hemoglobin (HbA1c), efficacy, and safety being well‐established,^3^, ^4^, ^5^ it became the recommended first‐line choice for patients with type 2 diabetes in 2005.^6^, ^7^ GLP‐1 RAs have been available as injectable formulations since exenatide received FDA approval in 2005 and as oral formulations since semaglutide was approved in 2019,^8^ trials have demonstrated significant weight and HbA1c reduction benefits of GLP‐1 RA compared with standard of care in patients with type 2 diabetes at high cardiovascular risk.^9^, ^10^ Post hoc studies from clinical studies have also shown the renal protective properties of GLP‐1 RA^11^ further supported by a meta‐analysis that demonstrated that GLP‐1 RA reduced worsening of kidney function in patients with type 2 diabetes.^12^ Conversely, metformin does not affect kidney function.^13^ An increase in the use of GLP‐1 RA as initial treatment in drug‐naive patients has been observed in Denmark^14^ regardless that no randomized clinical trial evidence has been published on how or whether GLP‐1 RA outperforms metformin in these patients. The rise in the use of GLP‐1 RA may partly stem from updates in guidelines and more in general due to emerging evidence of cardiorenal benefits of GLP‐1 RA. For example, the 2018 European Association for the Study of Diabetes (EASD) and American Diabetes Association consensus guidelines recommend a sodium‐glucose cotransporter‐2 (SGLT2) inhibitor or a GLP‐1 RA with proven cardiovascular benefits for patients with clinical cardiovascular disease.^15^ Additionally, the 2019 guidelines issued by the European Society of Cardiology and the EASD suggest using GLP‐1 RA as initial therapy in patients with type 2 diabetes and cardiovascular disease or those at high risk of cardiovascular disease (no class recommendation).^16^ Using the Danish nationwide registries, the purpose of this paper was to (1) compare the 1‐year risk of add‐on glucose‐lowering therapy and nonadherence among patients with prediabetes (HbA1c 42–47 mmol/mol) or diabetes (HbA1c ≥ 48 mmol/mol) in first‐line treatment with GLP‐1 RA or metformin. (2) Estimate the absolute change in HbA1c, estimated glomerular filtration rate (eGFR), and cholesterol levels during 1‐year treatment with GLP‐1 RA compared with treatment with metformin, in subgroups by whether the patient had prediabetes or diabetes to evaluate real‐world use.

## 2 METHODS

### 2.1 Data sources

In Denmark, all citizens have equal access to healthcare services, including both primary and hospital care. Danish residents are at birth or upon immigration assigned a unique 10‐digit personal identification number, making linkage of the national registers possible.^17^ The following seven registers were used for this study: (1) The Danish National Prescription Registry which holds information on dosage, dates, and anatomical therapeutic chemical (ATC) codes on all prescriptions dispensed from pharmacies since 1995^18^; (2) The Danish National Patient Registry, which holds information on all outpatient contacts and hospital admissions since 1977, coded according to The *International Classification of Disease* (ICD)^19^; (3) The Danish Civil Registration System, which holds information on the date of birth, sex, emigration/immigration, and vital status^20^; (4) The Income Statistics Registry which has data on yearly income^21^; (5) The Population Education Register which holds information on highest attained education^22^; (6) The Clinical Laboratory Information System Research Database, which holds information on individual‐level biomarker results and date and time of sampling^17^; and (7) The Danish Adult Diabetes Registry, which collects information on patients with diabetes from the primary and secondary health care sector including biomarker measurements.^23^

### 2.2 Study population and definition of treatment duration

Between January 1, 2018, and December 31, 2021, we identified incident (first‐line) users in monotherapy with GLP‐1 RA or metformin, having an HbA1c level above 42 mmol/mol (a list of ATC codes used for definitions is available in Table S1). Exclusion criteria included polycystic ovarian syndrome (defined as female sex and age under 40 years at the time of first metformin prescription), immigration within the last 10 years, age over 95 years, an eGFR measurement below 30 mL/min/1.73 m^2^ within the year before the index date, an ICD code indicating heart failure within the last 10 years (Table S2), missing prebaseline HbA1c data, an HbA1c level below 42 mmol/mol, or a diagnosis of gestational diabetes (DO244). The reason for excluding all patients with heart failure was due to our inability to determine the presence of congestive heart failure stage IV, which is contraindicated for GLP‐1 RA use. To ensure that the incident users in the study period were indeed new users, we reviewed all prescriptions dating back to the year 2000. Further, to ensure exclusive treatment with GLP‐1 RA or metformin, we incorporated a 90‐day grace period from the date of the initial prescription to evaluate the prescriptions of other glucose‐lowering drugs, only including those who claimed solely their initial prescription or more of the initial treatment GLP‐1 RA within this period. Patients were thus included 90 days after their first prescription (index). A patient was considered to be under treatment from the first prescription until the date of the subsequent prescription. If no additional prescription was obtained or the last prescription concluded, a treatment duration of 3 months was assumed. Metformin initiators were matched with GLP‐1 RA initiators in a ratio of 1:1 without replacement on sex, age (5‐year intervals), and latest HbA1c measurement within 1 year before initiation of antidiabetic treatment (42–47, 48–52, 53–57, 58–64, 65–74, >75 mmol/mol) to address concerns regarding exchangeability between the two groups. Only GLP‐1 RA initiators were included for whom a matched metformin initiator was available.

### 2.3 Outcomes

The following outcomes were evaluated: the 1‐year risk of add‐on glucose‐lowering therapy and the 1‐year risk of nonadherence. In patients with a baseline and second measurement during a 1‐year follow‐up, the changes in HbA1c, eGFR, and cholesterol levels were likewise evaluated.

#### 2.3.1 Definition of add‐on glucose‐lowering treatment and nonadherence

Add‐on therapy was defined as the initial prescription of an additional glucose‐lowering drug (including insulin) with GLP‐1 RA or metformin and the date hereof for those who were adherent to the initial treatment. Nonadherence was determined based on the date of treatment discontinuation with GLP‐1 RA or metformin, day 90 (index) if no further prescription than the initial was redeemed.

#### 2.3.2 Change in HbA1c, eGFR, and cholesterol levels

Biomarker values were assessed in posttreatment selected patients who were alive and had a measurement of the respective biomarker before and 3–12 months after treatment initiation, as the first available measurement within 12 months after treatment initiation. Among those who had a measurement, the absolute change was calculated as the difference to the last value available before treatment initiation in mmol/mol for HbA1c, mL/min/1.73 m^2^ for eGFR, and mmol/L for low‐density lipoprotein (LDL) cholesterol, and triglycerides. eGFR was calculated from the Chronic Kidney Disease Epidemiology Collaboration (CKD‐EPI) equation.^24^ Unreliable measurements were excluded as defined in Table S4.

### 2.4 Covariates

Age was calculated for the calendar year at treatment initiation; education level was defined as the highest achieved education level before treatment initiation and classified into four groups based on the International Standard Classification of Education (ISCED).^25^ Income was included as the equivalized income in the year before treatment initiation and categorized into quartiles for the main analysis.^26^ Comorbidities within 10 years before the treatment initiation were identified through the Danish National Prescription Registry and the Danish National Patient Registry (a list of ATC and diagnosis codes used for definitions is available in Table S2). Medicine use was identified through the Danish National Prescription Registry and defined as any redemption of a prescription 180 days before treatment initiation (a list of ATC codes is available in Table S3). Biomarkers were identified through the Clinical Laboratory Information System Research Database and the Danish Adult Diabetes Registry. Baseline values were included as the last value before treatment initiation, no longer than 1 year before.

### 2.5 Statistical methods

Baseline characteristics were summarized at the time of treatment initiation. Patients were followed from the index date until outcome, death, emigration, or December 31, 2022, whichever came first. All analyses were conducted in subgroups of HbA1c, that is, 42–48 mmol/mol indicating prediabetes and ≥ 48 mmol/mol indicating diabetes. The 1‐year probability of the outcomes, add‐on glucose‐lowering therapy, and nonadherence were calculated, using the empirical distribution function, as the number of patients with the respective outcome within *x* days after the index date divided by the number of patients at risk at that particular date. Biomarker measurements were analyzed in posttreatment selected patients who had a follow‐up measurement within 1 year, that is, excluding patients who died without follow‐up measurement and patients who were alive but did not have a follow‐up measurement registered. The baseline values and the absolute changes in biomarker measurements were summarized by interquartile ranges. Multiple logistic regression was used to estimate the standardized 1‐year risk, risk difference (average treatment effect among GLP‐1 RA initiators), and risk ratio (RR) using the g‐formula,^27^ where the reference population included all patients in the subgroups. Multiple linear regression was used to estimate the average changes of the biomarkers. All regression models were adjusted for additive effects of the baseline patient characteristics including co‐morbidity and co‐medicine but not for the matching variables (sex, age, and HbA1c levels). In the analysis of biomarkers, the baseline level of each specific biomarker was included in the regression analysis, except for HbA1c We repeated all analyses in subgroups of sex (male, female), age groups (<55 years, ≥55 years), previous cardiovascular disease, defined as previous stroke, ischemic heart disease, or peripheral vascular disease (present, absent), and hypertension (present, absent). We further performed the following supplementary analyses: The 1‐year probability of having a biomarker measurement during follow‐up was calculated as the number of patients with a measurement within *x* days after the index date divided by the number of patients at risk at that particular date. To describe the selection of patients for the biomarker analyses, we used multiple logistic regression to estimate the odds of having an HbA1c measurement during follow‐up. R version 4.2.1 was used for data management and analysis.^28^

## 3 RESULTS

**FIGURE 1:**
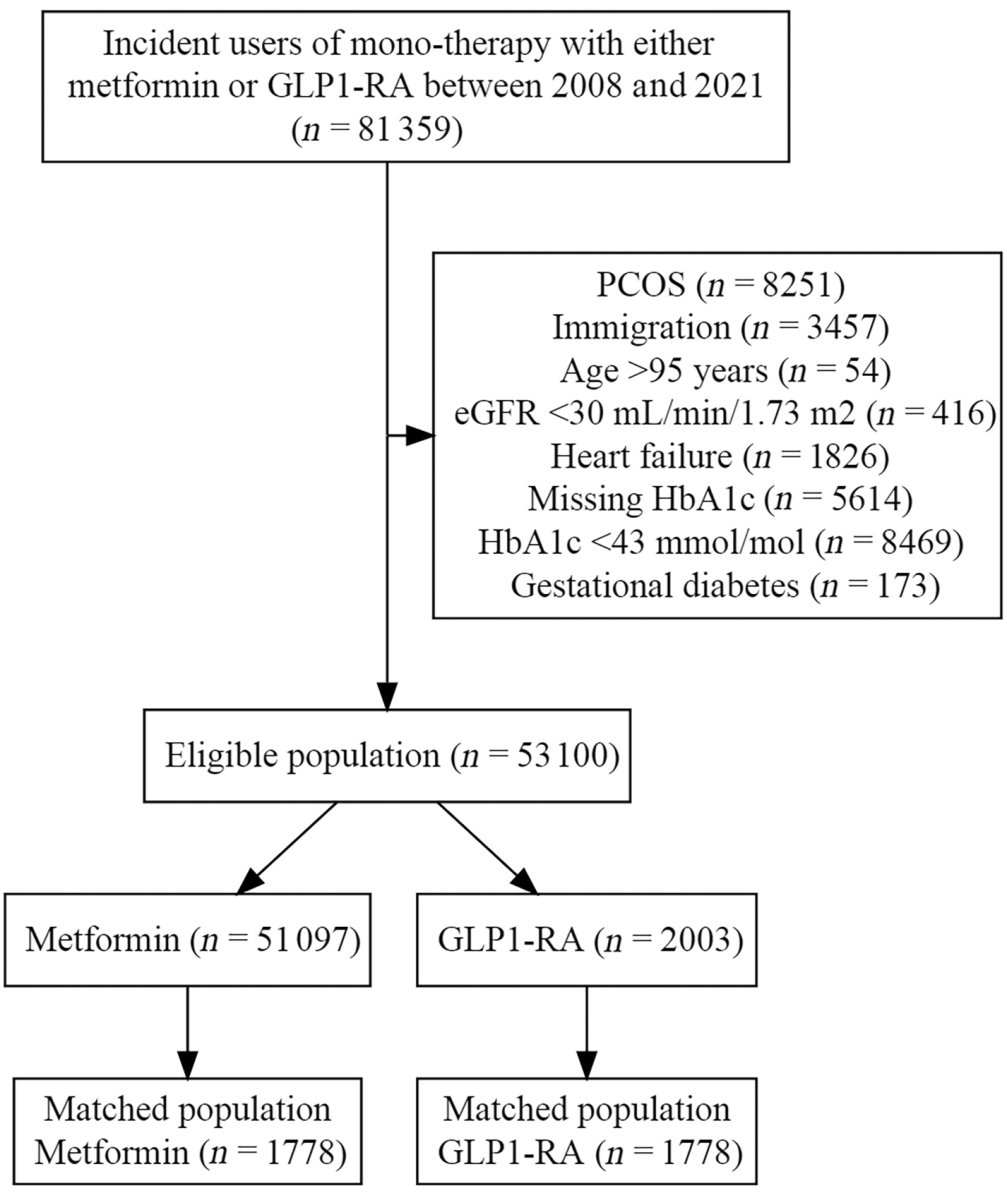
Flowchart. eGFR, estimated glomerular filtration rate; GLP‐1 RA, glucagon‐like peptide‐1 receptor agonist; HbA1c, glycosylated hemoglobin; PCOS, polycystic ovary syndrome.

Between January 1, 2018, and December 31, 2021, we identified 81 359 first‐line monotherapy users initiating treatment with either GLP‐1 RA or metformin. A total of 28 259 were excluded for the following reasons; polycystic ovarian syndrome, immigration 10 years prior, age over 95 years, eGFR below 30 mL/min/1.73 m^2^, heart failure, missing prebaseline HbA1c, HbA1c level below 42 mmol/mol, or gestational diabetes. Of the 53 100 eligible for inclusion, 2003 were identified as GLP‐1 RA initiators, and 51 097 were identified as metformin users. For 225 of the GLP‐1 RA initiators, no matching metformin initiator was available. These GLP‐1 RA initiators were not included, resulting in a final study population of 1778 GLP‐1 RA and metformin initiators, respectively (Figure 1).

### 3.1 Baseline characteristics

Compared with metformin initiators, GLP‐1 RA initiators had higher levels of education and higher incomes. The degree of urbanization was similar in the two treatment groups. Similar distributions were found for comorbidities. In regard to medical therapy before treatment initiation, GLP‐1 RA initiators more often redeemed prescriptions for mineralocorticoids, beta‐blockers, calcium channel blockers, renin‐angiotensin system inhibitors, loop diuretics, and thiazides, but less often for acetylsalicylic acids and statins, compared with metformin initiators. Similar distributions for baseline biomarkers were found (Table 1). Among those initiating GLP‐1 receptor agonists, 57.5% started with liraglutide, 42.0% with semaglutide, and 0.5% with dulaglutide.

**TABLE 1:** Baseline characteristics.

Variable	Level	Metformin (*n* = 1778)	GLP‐1 RA (*n* = 1778)	Total (*n* = 3556)
Age (years)	Median [IQR]	58 [51–65]	58 [51–65]	58 [51–65]
Sex, *n* (%)	Male	811 (45.6)	811 (45.6)	1622 (45.6)
Educational level, *n* (%)	Basic education	639 (35.9)	504 (28.3)	1143 (32.1)
	General upper secondary education	787 (44.3)	811 (45.6)	1598 (44.9)
	Bachelor‐level education	307 (17.3)	376 (21.1)	683 (19.2)
	Masters or PhD	45 (2.5)	87 (4.9)	132 (3.7)
Income (DKK^a^)	Median [IQR]	198 734.8 [156 070–262 958]	237 261.2 [183 174–299 376]	219 009 [165 321–282 185]
Degree of urbanization, *n* (%)	Rural	685 (38.5)	636 (35.8)	1321 (37.1)
	Suburb	586 (33.0)	608 (34.2)	1194 (33.6)
	Urban	507 (28.5)	534 (30.0)	1041 (29.3)
Index year	2018	448 (25.7)	83 (4.8)	531 (15.2)
	2019	430 (24.7)	158 (9.1)	588 (16.9)
	2021	412 (23.6)	380 (21.8)	792 (22.7)
	2022	453 (26.0)	1122 (64.4)	1575 (45.2)
Biomarkers				
Hba1c (mmol/mol), *n* (%)	42,47	1220 (68.6)	1221 (68.7)	2441 (68.6)
	48, 52	359 (20.2)	361 (20.3)	720 (20.2)
	53, 57	100 (5.6)	98 (5.5)	198 (5.6)
	>=58	99 (5.6)	98 (5.5)	197 (5.5)
Estimated glomerular filtration rate (mL/min/1.73 m^2^), *n* (%)	30, 44	42 (3.6)	49 (5.0)	91 (4.2)
	45, 59	221 (19.1)	220 (22.3)	441 (20.5)
	60, 89	652 (56.2)	526 (53.2)	1178 (54.8)
	>=90	245 (21.1)	193 (19.5)	438 (20.4)
	Missing	618	790	1408
Low‐density lipoprotein cholesterol (mmol/L), *n* (%)	<=2.2	434 (36.9)	328 (31.8)	762 (34.5)
	2.3, 3.1	391 (33.2)	359 (34.9)	750 (34.0)
	3.2, 4.1	259 (22.0)	254 (24.7)	513 (23.3)
	[4.2, 5.1	80 (6.8)	75 (7.3)	155 (7.0)
	>=5.2	12 (1.0)	14 (1.4)	26 (1.2)
	Missing	602	748	1350
Triglycerides (mmol/L), *n* (%)	<=1.7	654 (38.8)	614 (36.7)	1268 (37.7)
	1.8, 2	215 (12.7)	221 (13.2)	436 (13.0)
	2.1, 6	789 (46.8)	800 (47.8)	1589 (47.3)
	>=6.1	29 (1.7)	39 (2.3)	68 (2.0)
	Missing	91	104	195
Comorbidities				
Hypertension, *n* (%)		292 (16.4)	314 (17.7)	606 (17.0)
Ischemic heart disease, *n* (%)		170 (9.6)	181 (10.2)	351 (9.9)
Stroke, *n* (%)		101 (5.7)	81 (4.6)	182 (5.1)
Peripheral vascular disease, *n* (%)		17 (1.0)	13 (0.7)	30 (0.8)
Renal disease, *n* (%)		20 (1.1)	35 (2.0)	55 (1.5)
Atrial fibrillation, *n* (%)		69 (3.9)	80 (4.5)	149 (4.2)
Cancer, *n* (%)		123 (6.9)	93 (5.2)	216 (6.1)
Chronic obstructive pulmonary disease, *n* (%)		59 (3.3)	44 (2.5)	103 (2.9)
Concomitant medication collected within 180 days before treatment initiation				
Mineralocorticoid receptor antagonists, *n* (%)		78 (4.4)	103 (5.8)	181 (5.1)
Beta blockers, *n* (%)		318 (17.9)	365 (20.5)	683 (19.2)
Renin‐angiotensin‐system acting agents, *n* (%)		779 (43.8)	831 (46.7)	1610 (45.3)
Loop diuretics, *n* (%)		124 (7.0)	264 (14.8)	388 (10.9)
Calcium channel blockers, *n* (%)		373 (21.0)	419 (23.6)	792 (22.3)
Thiazide diuretics, *n* (%)		218 (12.3)	274 (15.4)	492 (13.8)
Acetylsalicylic acid, *n* (%)		221 (12.4)	199 (11.2)	420 (11.8)
Statins, *n* (%)		734 (41.3)	647 (36.4)	1381 (38.8)

Abbreviations: DKK, Danish Krone; GLP‐1 RA, gucagon‐like peptide‐1 receptor agonist; HbA1c, glycosylated hemoglobin; IQR, interquartile range.

^a^ 100 DKK approximates 13.42 Euro.

### 3.2 Add‐on therapy and nonadherence

**FIGURE 2:**
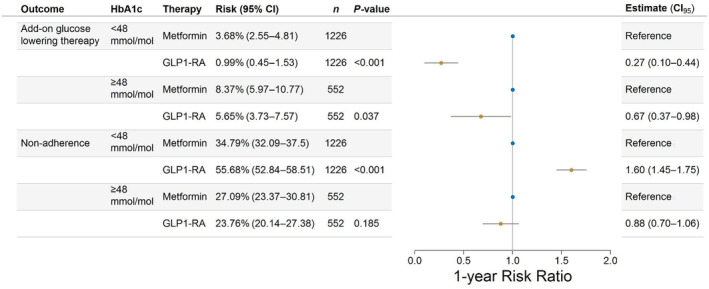
Absolute risk and risk ratio of first‐line monotherapy glucagon‐like peptide‐1 receptor agonist (GLP‐1 RA) initiation on add‐on glucose‐lowering therapy and nonadherence, respectively, within 1 year of treatment initiation, compared with first‐line monotherapy metformin initiation. CI, confidence interval; HbA1c, glycosylated hemoglobin.

The 1‐year absolute risks of add‐on glucose‐lowering therapy stratified according to baseline HbA1c are shown in Figure S1A. After standardizing with respect to the relevant confounders, estimating the average treatment effects among the treated, GLP‐1 RA initiation in those with prediabetes was associated with a 1‐year risk of add‐on glucose‐lowering therapy of 0.99% (95% confidence interval [CI]: 0.45–1.53), whereas metformin initiation was associated with a risk of add‐on glucose‐lowering therapy of 3.68% (95% CI: 2.55–4.81), resulting in a RR of 0.27 (95% CI: 0.10–0.44, *p*‐value<0.001). In patients with diabetes, GLP‐1 RA was associated with an RR of 0.67 (95% CI: 0.37–0.98, *p*‐value = 0.037) (Figure 2). Among those initiating add‐on therapy, 40.7% of metformin users added SGLT2 inhibitors, 36.0% added GLP‐1 receptor agonists (RAs), 14.0% added dipeptidyl peptidase 4 (DPP‐4) inhibitors, 6% added insulin, and 3.3% added sulfonylureas. For GLP‐1 RA initiators, 84.8% added metformin, 13.0% added SGLT2 inhibitors, and 2.2% added insulin, with no additions of DPP‐4 inhibitors or sulfonylureas. For nonadherence, the 1‐year absolute risks of nonadherence stratified according to baseline HbA1c are shown in Figure S1B. After standardizing with respect to the relevant confounders and estimating the average treatment effects among the treated, GLP‐1 RA initiation in patients with prediabetes was associated with a 1‐year risk of 55.68% (95% CI: 52.84–58.51) of nonadherence, whereas the risk associated with metformin initiation was 34.79% (95% CI: 32.09–37.50), yielding a RR of 1.60 (95% CI: 1.45;1.75, *p* < 0.001) for GLP‐1 RA initiation compared to metformin initiation. For patients with diabetes, this RR was 0.88 (95% CI: 0.70–1.06, *p* = 0.185) (Figure 2). Among those who did not adhere to their initial treatment within the 1‐year follow‐up period, 90.5% of metformin initiators and 94.2% of GLP1‐RA initiators did not start another glucose‐lowering therapy afterward. For the risk of add‐on therapy, the magnitude but not the direction changed in subgroups of sex, age, cardiovascular disease (CVD), and hypertension. Likewise, in all subgroups, the risk of nonadherence was increased for GLP‐1 RA initiation, compared with metformin initiation (Figure S2).

### 3.3 Differences in biochemical markers

**FIGURE 3:**
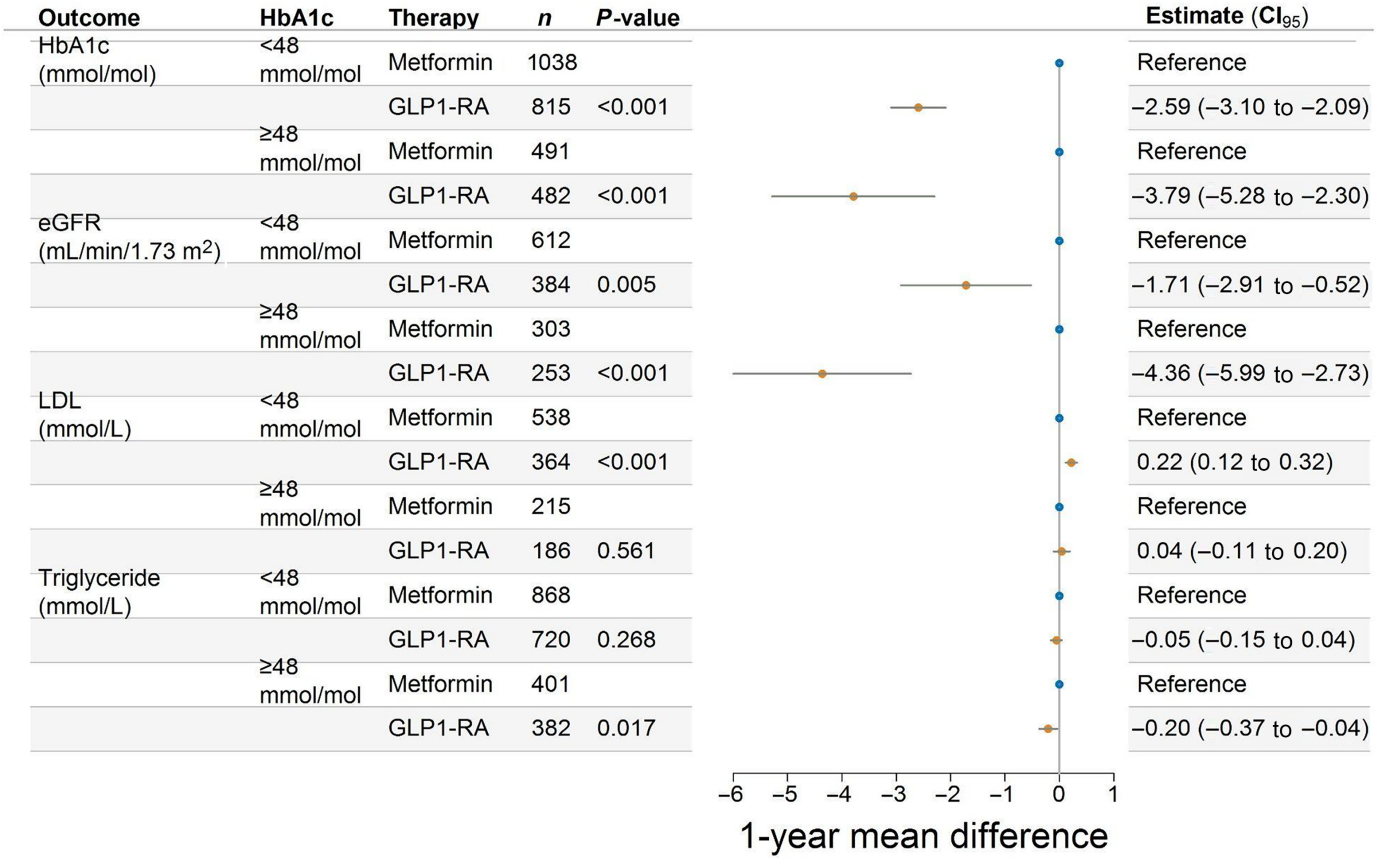
Average difference in the effect of first‐line monotherapy glucagon‐like peptide‐1 receptor agonist (GLP‐1 RA) initiation compared with first‐line monotherapy metformin initiation on glycosylated hemoglobin (HbA1c), estimated glomerular filtration rate (eGFR), low‐density lipoprotein (LDL) cholesterol, and triglycerides. CI, confidence interval.

Investigating the differences in biomarkers, we included only those with a measurement during follow‐up. Among GLP‐1 RA initiators, these percentages were 73% (HbA1c), 36% (eGFR), 31% (LDL cholesterol), and 62% (triglycerides). For metformin initiators, the corresponding percentages were 86%, 51%, 42%, and 71%. There were the following unadjusted mean differences (SD) in biomarkers for treatment with GLP‐1 RA versus metformin: HbA1c; −6.43 mmol/mol (8.95) versus −2.95 mmol/mol (8.39), eGFR; −2.40 mL/min/1.73 m^2^ (10.03) versus 0.61 mL/min/1.73 m^2^ (9.06), LDL cholesterol; −0.22 mmol/L (0.71) versus −0.31 mmol/L (0.93), and triglycerides; −0.37 mmol/L (1.25) versus −0.21 mmol/L (1.19), respectively. In groups defined by baseline values, larger improvements were seen on the absolute scale in patients with a high baseline value compared to patients with a low baseline value (Figures S3–S6). After adjusting for relevant confounders, GLP‐1 RA initiation was associated with a greater average reduction in HbA1c, eGFR, and triglycerides compared with metformin initiation in both patients with prediabetes and patients with diabetes. A small increase in LDL cholesterol compared to metformin initiation was observed (Figure 3). The overall direction and magnitudes of average changes associated with GLP‐1 RA initiation compared to metformin initiation, on HbA1c, eGFR, LDL cholesterol, and triglycerides did not change in subgroups of sex, age, CVD, and hypertension (Figure S7).

### 3.4 Supplementary analyses

A larger proportion of the patients initiating metformin died compared to patients initiating GLP‐1 RA, 16 and ≤5, respectively. There was no loss to follow‐up, and none emigrated during follow‐up. GLP‐1 RA users had relatively lower odds of having an HbA1c measurement during follow‐up (odds ratio: 0.41, 95% CI: 0.35–0.50), compared with metformin users, when adjusting for sex, age, education, income, degree of urbanization, co‐morbidities, and co‐medicine (Table S5). The baseline characteristics by status of prediabetes or diabetes revealed similar distributions as Table 1 (Table S6). The tendencies in the timing of biomarker measurement were similar regardless of treatment, although metformin initiators had a higher probability of having their biomarker levels measured in general (Figure S8).

## 4 DISCUSSION

In this study, estimating the average treatment effect among the treated, first‐line GLP‐1 RA initiation was associated with a lower 1‐year risk of requiring additional glucose‐lowering treatment in both patients with prediabetes and diabetes, compared with starting with metformin. However, GLP‐1 RA initiation was also associated with an increased risk of nonadherence in patients with prediabetes, with no difference in patients with diabetes, compared with metformin initiation. Regardless of diabetes status, first‐line GLP‐1 RA initiation was significantly associated with improved HbA1c control; however, larger reductions in eGFR and less decreased LDL cholesterol levels during 1 year of follow‐up, compared with first‐line metformin initiation.

The reported discontinuation rates and use of rescue medication in GLP‐1 RA trials are overall lower than the estimates reported in our study. The reported discontinuation rates ranged from 3% to 13.7%, and the reported use of rescue medication ranged from 5% to 13%, albeit higher rates of discontinuation and use of rescue medication were observed in trials with longer duration.^9^, ^29^, ^30^, ^31^ In observational studies based on European and American study populations, however, the reported rates of discontinuation and add‐on therapy were much higher than reported in the randomized clinical trials. During the first year of treatment, up to 74% of patients receiving GLP‐1 RA reported a change in treatment either due to up‐titration, complete cessation of medication, or augmentation. Complete discontinuation was observed in 64% of the patients and augmentation occurred in 17% of the patients.^32^ Similarly, a study carried out in the United Kingdom showed a discontinuation rate of 45% and 65% at 12 and 24 months, respectively.^33^ These observational findings align with real‐world adherence to other glucose‐lowering medications.^34^ Our estimates depended on prediabetes or diabetes baseline status. Trials excluded patients with prediabetes, and the abovementioned observational studies included patients with higher HbA1c levels at baseline, affecting the comparability. However, our estimates in patients with diabetes align with the reported estimates from the randomized trials. Reasons for our high estimates of nonadherence in patients with prediabetes could be that gastrointestinal adverse effects have been reported in association with GLP‐1 RA treatment, and it is reasonable to speculate that patients with prediabetes might exhibit a reduced willingness to tolerate these effects.^35^, ^36^ Despite the high risk of nonadherence, patients with prediabetes and in GLP‐1 RA treatment were not associated with an increased risk of add‐on glucose‐lowering treatment, which could be indicative of an effective weight reduction and control of the HbA1c simply weight reduction, or adverse effects. Patients with diabetes and in treatment with GLP‐1 RA were not associated with a significantly lower risk of add‐on glucose‐lowering treatment compared to metformin initiators. Further studies with longer follow‐up times and larger sample sizes are needed to further evaluate this outcome.

The observed changes in the biochemical markers in this study were similar but smaller in magnitude than reported in randomized trials.^29^, ^30^, ^31^, ^37^, ^38^, ^39^ Similarly, in the semaglutide treatment effect in people with obesity trial, a larger reduction in HbA1c was observed in the semaglutide group compared with placebo, with a decrease of −0.29 (95% CI: −0.32 to −0.26). Additionally, there were relative reductions in LDL cholesterol and triglycerides by 68 weeks, with values of 0.96 (95% CI: 0.94–0.98) and 0.84 (95% CI: 0.81–0.87), respectively.^40^ Reasons for discrepancies in magnitudes of effect could be that trial participants were generally enrolled with a higher baseline HbA1c, higher level of dysregulation, and longer diabetes duration, which could cause higher absolute differences between baseline achieved HbA1c levels at the end of the trial. Trial participants were also regularly checked and evaluated by doctors to ensure the optimal treatment to reach treatment targets, which was not present in our cohort. But, most importantly, we imposed a selection bias in our study population as we only were able to observe a change among those with a second measurement during follow‐up, and the results must be interpreted with this in mind.

### 4.1 Strengths and limitations

There are several strengths of this study, including its large sample size and the nationwide coverage of data. Additionally, this study utilized information on morbidities and medication from officially maintained registers for administrative purposes, providing a reliable source of data, without relying on self‐reporting. One potential limitation of this study is the observational design, which entails a risk of residual confounding. Specifically, we lacked information on factors, such as body mass index and indication for use, which could have influenced the results. Additionally, other potential confounders, such as factors affecting the physician's prescription preferences, were not accounted for and we cannot rule out confounding by indication. Selection bias may have affected the generalizability of our findings. In particular, many individuals were excluded due to a lack of information on their HbA1c levels before treatment initiation. This may in part be attributed to the fact that some GLP‐1 RA products are used to treat obesity, which is a target group beyond the scope of this paper. Furthermore, GLP‐1 RA initiators may be a selected affluent group, since the cost of this therapy is higher than metformin. However, in Denmark, medication is subsidized by the government; thus, the maximum copayment of any patient amounts to approximately 595 Euro per year (in 2023),^38^, ^41^ thereby minimizing this risk slightly. We note that the effect estimates should be interpreted considering the matched population which imposed a selection. Furthermore, those who died during follow‐up could have been misclassified, that is, as nonadherent; however, mortality was rare. We evaluated the biomarker outcomes in the period 3 to 12 months after treatment initiation and analyzed only patients who had a second measurement in that period. The posttreatment selection of patients for the biomarker analyses was thus affected by the timing of follow‐up measurements, reflecting disease progression and patient behavior, limiting the interpretation of the changes in biomarker values. Hence, our results have a hypothesis‐generating character. Notably, nonadherent patients with subsequent blood chemistry measurements may not reflect the treatment's potential benefits accurately, while patients receiving add‐on treatment could contribute with blood chemistry measurements affected by other treatments.

### 4.2 Conclusion

In patients with prediabetes, GLP‐1 RA initiation was associated with a lower risk of requiring additional glucose‐lowering therapy, compared with metformin; however, metformin initiation was associated with substantially higher levels of adherence. In patients with diabetes, GLP‐1 RA initiation was also associated with a lower risk of requiring additional glucose‐lowering therapy, but no difference in the risk of nonadherence, compared to metformin initiation.

## 5 ETHICS

Danish registry‐based studies performed for the sole purpose of statistics and scientific research do not require patient consent nor ethical committee approval, as stated in The Danish Data Protection Act. Approval to use the data sources for research purposes was granted by the data responsible institute of the Capital Region of Denmark (approval number P‐2019‐393) under the General Data Protection Regulation (GDPR). Data are accessed on secure servers under Statistics Denmark and cannot be shared, according to Danish legislation.^42^

## AUTHOR CONTRIBUTIONS

KKS contributed to conceptualization, methodology, data curation, formal analysis, and original draft preparation. TAG contributed to designing the methodology and data analysis. LK, ELF, HEP, ALM, MPA, and UPB contributed to critical review of the manuscript, data interpretation, and manuscript editing. CT‐P contributed to critical review of the manuscript, data interpretation, manuscript editing, project administration, and funding acquisition. BZ contributed to manuscript editing, manuscript review, conceptualization, methodology, data curation, and supervision. The authors collectively discussed the research design, and critically reviewed and edited the manuscript for intellectual content.

## FUNDING INFORMATION

The authors received no financial support for this article's research, authorship, or publication.

## CONFLICT OF INTEREST STATEMENT

CT‐P reports grants from Bayer and Novo Nordisk unrelated to the current study. UPB has served on advisory boards for Novo Nordisk, Sanofi, and Vertex and has received lecture fees from Novo Nordisk and Sanofi. LK reports speakers' honorariums from AstraZeneca, Bayer, Boehringer, Novartis, and Novo. All other authors declare no competing interests.

## Supporting information


**Data S1.** Supporting Information.
