# Supplementary material for: Comparing Glucagon‐like peptide‐1 receptor agonists versus metformin in drug‐naive patients: A nationwide cohort study
Source: J Diabetes. 2024 Oct 4;16(10):e70000. doi: 10.1111/1753-0407.70000 (PMC11450598; doi:10.1111/1753-0407.70000)

# Supplementum

Table S1: ATC codes to define GLP-1 RA and metformin groups

| **Drug type** | **Active substance** | **ATC-code** |
| --- | --- | --- |
| Metformin | metformin hydrochloride | A10BA02 |
| GLP-1 RA | exenatide | A10BJ01 |
|  | semaglutide | A10BJ06 |
|  | dulaglutide | A10BJ05 |
|  | liraglutide | A10BJ02 |

Table S2: Codes to define comorbidities

| **Disease** |  | **ICD-10** | **ATC code** |
| --- | --- | --- | --- |
| Heart failure |  | I110, I130, I132, I420, I426-I429, I50 |  |
| Hypertension |  | I10, I109, I11, I110, I119, I119A, I12, I120, I129, I13, I130, I131, I132, I139, I15, I150, I151, I152, I158, I159 | CO2A, CO2B, CO2C, CO2DA, CO2DB, CO2DD, CO2DG, CO2L, CO3A, CO3B, CO3D, CO3E, CO3X, CO7A, CO7B, CO7C, CO7D, CO7F, CO8, CO9AA, CO9BA, CO9BB, CO9CA, CO9DA, CO9DB, CO9XA02, CO9XA52 |
| Ischemic heart disease |  | I20, I23, I24, I25 |  |
| Stroke |  | I60-61, I63-64, G45 |  |
| Peripheral vascular disease |  | I70, I739 |  |
| Renal disease |  | N02-08, N11-12, N14, N17-19, N26, N158-160, N162-164, N168, Q612-613, Q615, Q619, E112, E131, E142, I120, R34 |  |
| Atrial fibrillation |  | I48 |  |
| Cancer |  | C00-C97 |  |
| Chronic obstructive pulmonary disease |  | J42, J44 |  |

Table S3: ATC codes to define concomitant medication

| **Drug** | **ATC code** |
| --- | --- |
| Mineralocorticoids | C03D |
| Beta-blocker | C07 |
| Renin-angiotensin system inhibitor | C09 |
| Loop diuretics | C03C, C03EB |
| Calcium channel blockers | C08 |
| Thiazide | CO3A |
| Acetylsalicylic acid | B01AC06, NO2BA01 |
| Statin | C10A, A10BH51, A10BH52 |

Table S4: Possible ranges for biomarker test, outliers awarded NA

| **Blood chemistry test** | **Minimum** | **Maximum** |
| --- | --- | --- |
| HbA1c (mmol/mol) | 0.2 | 120 |
| Creatinine (µmol/L) | 10 | 1800 |
| LDL cholesterol | 0.52 | 6.68 |
| Triglycerides | 0.11 | 12.43 |

Table S5: Logistic regression with having a follow-up HbA1c measurement as the outcome

| **Variable** | **Level** | **Odd ratio (95% CI)** | **P-value** |
| --- | --- | --- | --- |
| Treatment | Metformin | Reference | |
|  | GLP-1 RA | 0.41 (0.35;0.5) | <0.001 |
| Age (years) | <=45 | Reference |  |
|  | (45,50] | 1.03 (0.73;1.44) | 0.87 |
|  | (50,55] | 0.96 (0.7;1.31) | 0.787 |
|  | (55,60] | 1.05 (0.76;1.45) | 0.77 |
|  | (60,65] | 1.27 (0.9;1.78) | 0.176 |
|  | (65,70] | 1.3 (0.87;1.93) | 0.197 |
|  | (70,75] | 1.27 (0.83;1.96) | 0.276 |
|  | (75,80] | 1.51 (0.85;2.68) | 0.163 |
| Sex | Female | Reference |  |
|  | Male | 0.88 (0.74;1.05) | 0.171 |
| Educational level | Basic education | Reference |  |
|  | General upper secondary education | 1.03 (0.84;1.27) | 0.75 |
|  | Bachelor level education | 1.05 (0.81;1.36) | 0.726 |
|  | Masters or PhD | 1.02 (0.65;1.6) | 0.93 |
| Income (DKK) | < 174,000] | Reference |  |
|  | (174,000e - 224,000] | 1.03 (0.8;1.32) | 0.822 |
|  | (224,000 - 305,000] | 1.15 (0.9;1.47) | 0.253 |
|  | > 305,000 | 0.95 (0.72;1.26) | 0.73 |
| Degree of urbanization | Rural | Reference |  |
|  | Suburb | 0.86 (0.7;1.06) | 0.168 |
|  | Urban | 0.65 (0.52;0.8) | <0.001 |
| Hba1c (mmol/mol) | 42-47 | Reference |  |
|  | 48-53 | 2.32 (1.8;2.98) | <0.001 |
|  | >53 | 2.68 (1.92;3.73) | <0.001 |
| Hypertension yes, no as reference | | 1.02 (0.78;1.34) | 0.886 |
| Ischemic heart disease yes, no as reference | | 1.16 (0.81;1.65) | 0.427 |
| Stroke yes, no as reference | | 0.77 (0.51;1.16) | 0.208 |
| Peripheral vascular disease yes, no as reference | | 0.41 (0.17;1.02) | 0.055 |
| Renal disease yes, no as reference | | 1.33 (0.63;2.8) | 0.462 |
| Atrial fibrillation yes, no as reference | | 1 (0.61;1.62) | 0.984 |
| Cancer yes, no as reference | | 0.83 (0.58;1.2) | 0.33 |
| Chronic obstructive pulmonary disease yes, no as reference | | 0.87 (0.51;1.48) | 0.603 |
| Aldosterone receptor antagonists yes, no as reference | | 1.26 (0.81;1.96) | 0.312 |
| Beta blockers yes, no as reference | | 1.17 (0.9;1.52) | 0.236 |
| Renin-angiotensin-system acting agents yes, no as reference | | 1.1 (0.9;1.33) | 0.348 |
| Loop diuretics yes, no as reference | | 1.28 (0.94;1.72) | 0.113 |
| Calcium channel blockers yes, no as reference | | 1.01 (0.81;1.27) | 0.906 |
| Thiazide diuretics yes, no as reference | | 1.25 (0.96;1.64) | 0.102 |
| Acetylsalicylic acid yes, no as reference | | 1.19 (0.84;1.67) | 0.329 |
| Statins yes, no as reference | | 1.36 (1.11;1.67) | 0.003 |

Table S6: Baseline characteristics by status of pre-diabetes or diabetes at initiation

|  |  | Pre-diabetes (HbA1c 42-47 mmol/mol) | | Diabetes (HbA1c >47 mmol/mol) | |
| --- | --- | --- | --- | --- | --- |
| Variable | Level | Metformin (n=1778) | GLP-1 RA (n=1778) | Metformin (n=1778) | GLP-1 RA (n=1778) |
| Age (years) | Median [IQR] | 58 [52, 65] | 58 [52, 64] | 58 [50, 66] | 57 [50, 66] |
| Sex, n (%) | Male | 534 (44.4) | 534 (44.4) | 259 (48.0) | 259 (48.0) |
| Educational level, n (%) | Basic education | 440 (36.6) | 332 (27.6) | 189 (35.0) | 160 (29.6) |
|  | General upper secondary education | 534 (44.4) | 561 (46.6) | 239 (44.3) | 236 (43.7) |
|  | Bachelor level education | 195 (16.2) | 252 (20.9) | 103 (19.1) | 116 (21.5) |
|  | Masters or PhD | 34 (2.8) | 58 (4.8) | 9 (1.7) | 28 (5.2) |
| Income (DKK*) | Median [IQR] | 199,333.1 [154,591.0, 261,173.3] | 245,211.2 [189,454.3, 307,687.4] | 196,488.8 [159,012.3, 264,133.6] | 221,072 [169,172.3, 280,187.8] |
| Degree of urbanization, n (%) | Rural | 460 (38.2) | 384 (31.9) | 210 (38.9) | 237 (43.9) |
|  | Suburb | 382 (31.8) | 439 (36.5) | 188 (34.8) | 160 (29.6) |
|  | Urban | 361 (30.0) | 380 (31.6) | 142 (26.3) | 143 (26.5) |
| Index year | 2018 | 331 (27.5) | 61 (5.1) | 117 (21.7) | 22 (4.1) |
|  | 2019 | 311 (25.9) | 112 (9.3) | 119 (22.0) | 46 (8.5) |
|  | 2021 | 259 (21.5) | 241 (20.0) | 153 (28.3) | 139 (25.7) |
|  | 2022 | 302 (25.1) | 789 (65.6) | 151 (28.0) | 333 (61.7) |
| **Comorbidities** | | | | |  |
| Hypertension, n (%) | | 314 (26.1) | 302 (25.1) | 120 (22.2) | 167 (30.9) |
| Ischemic heart disease, n (%) | | 169 (14.0) | 159 (13.2) | 72 (13.3) | 85 (15.7) |
| Stroke, n (%) | | 82 (6.8) | 71 (5.9) | 40 (7.4) | 32 (5.9) |
| Peripheral vascular disease, n (%) | | 39 (3.2) | 21 (1.7) | 17 (3.1) | 23 (4.3) |
| Renal disease, n (%) | | 26 (2.2) | 29 (2.4) | 10 (1.9) | 31 (5.7) |
| Atrial fibrillation, n (%) | | 56 (4.7) | 63 (5.2) | 27 (5.0) | 38 (7.0) |
| Cancer, n (%) | | 110 (9.1) | 91 (7.6) | 50 (9.3) | 47 (8.7) |
| Chronic obstructive pulmonary disease, n (%) | | 67 (5.6) | 66 (5.5) | 33 (6.1) | 26 (4.8) |
| **Concomitant medication collected within 180 days before treatment initiation** | | | | |  |
| Mineralocorticoid Receptor Antagonists, n (%) | | 61 (5.1) | 59 (4.9) | 9 (1.7) | 32 (5.9) |
| Beta blockers, n (%) | | 215 (17.9) | 222 (18.5) | 90 (16.7) | 129 (23.9) |
| Renin-angiotensin-system acting agents, n (%) | | 530 (44.1) | 554 (46.1) | 227 (42.0) | 253 (46.9) |
| Loop diuretics, n (%) | | 93 (7.7) | 155 (12.9) | 26 (4.8) | 92 (17.0) |
| Calcium channel blockers, n (%) | | 246 (20.4) | 281 (23.4) | 119 (22.0) | 132 (24.4) |
| Thiazide diuretics, n (%) | | 152 (12.6) | 195 (16.2) | 60 (11.1) | 76 (14.1) |
| Acetylsalicylic acid, n (%) | | 143 (11.9) | 116 (9.6) | 67 (12.4) | 68 (12.6) |
| Statins, n (%) | | 504 (41.9) | 419 (34.8) | 210 (38.9) | 207 (38.3) |

*100 DKK approximates 13.42 Euro

Figure S1: (A) The frequency of add-on second-line glucose-lowering therapy to the first-line therapy (GLP-1 RA or metformin) by baseline HbA1c (Pre-diabetes: <48 or Diabetes: >=48 mmol/mol) and (B) The frequency of non-adherence of first-line glucose-lowering therapy (GLP-1 RA or metformin) by baseline HbA1c (Pre-diabetes: <48 or Diabetes: >=48 mmol/mol).


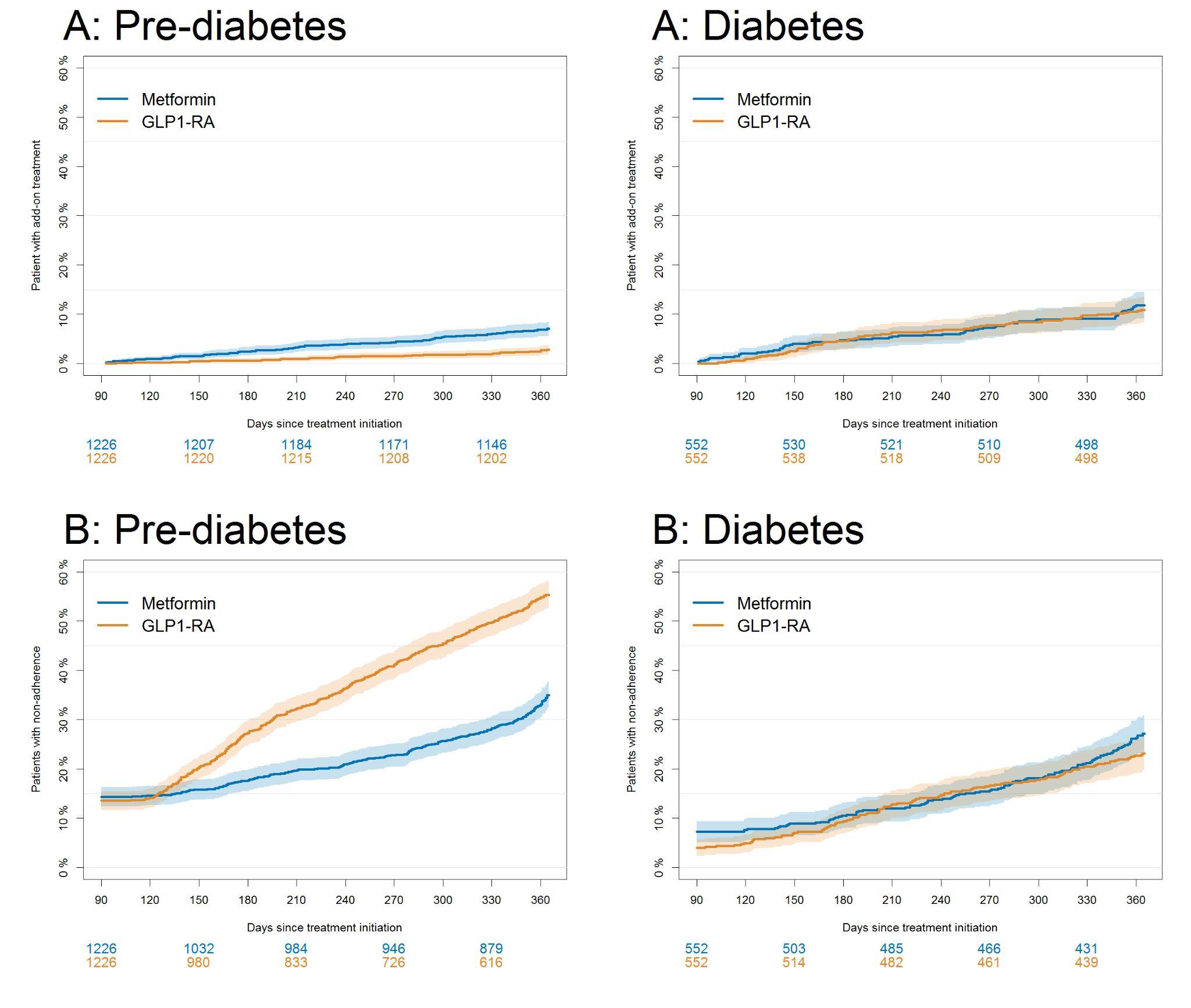


Figure S2: Risk ratio of add-on glucose-lowering treatment and non-adherence for first-line initiation of GLP-1 RA versus metformin, in subgroups of sex, age, previous cardiovascular disease (stroke, ischemic heart disease, or peripheral vascular disease), and hypertension


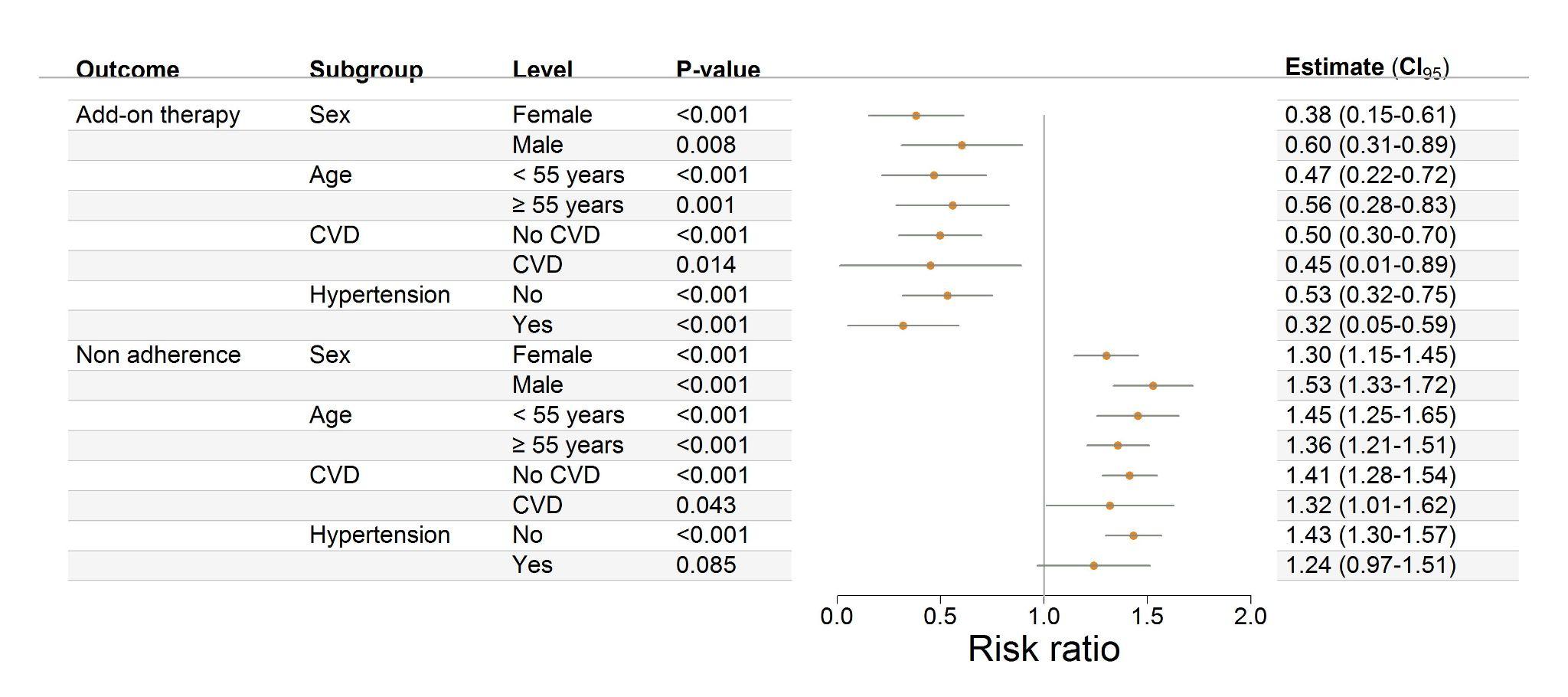


Figure S3: Difference between baseline HbA1c (mmol/mol) and first available measurement during one-year follow-up, by baseline value of HbA1c. Embedded box plot indicating the median and interquartile range. Metformin; n=1529, GLP-1 RA; n=1297
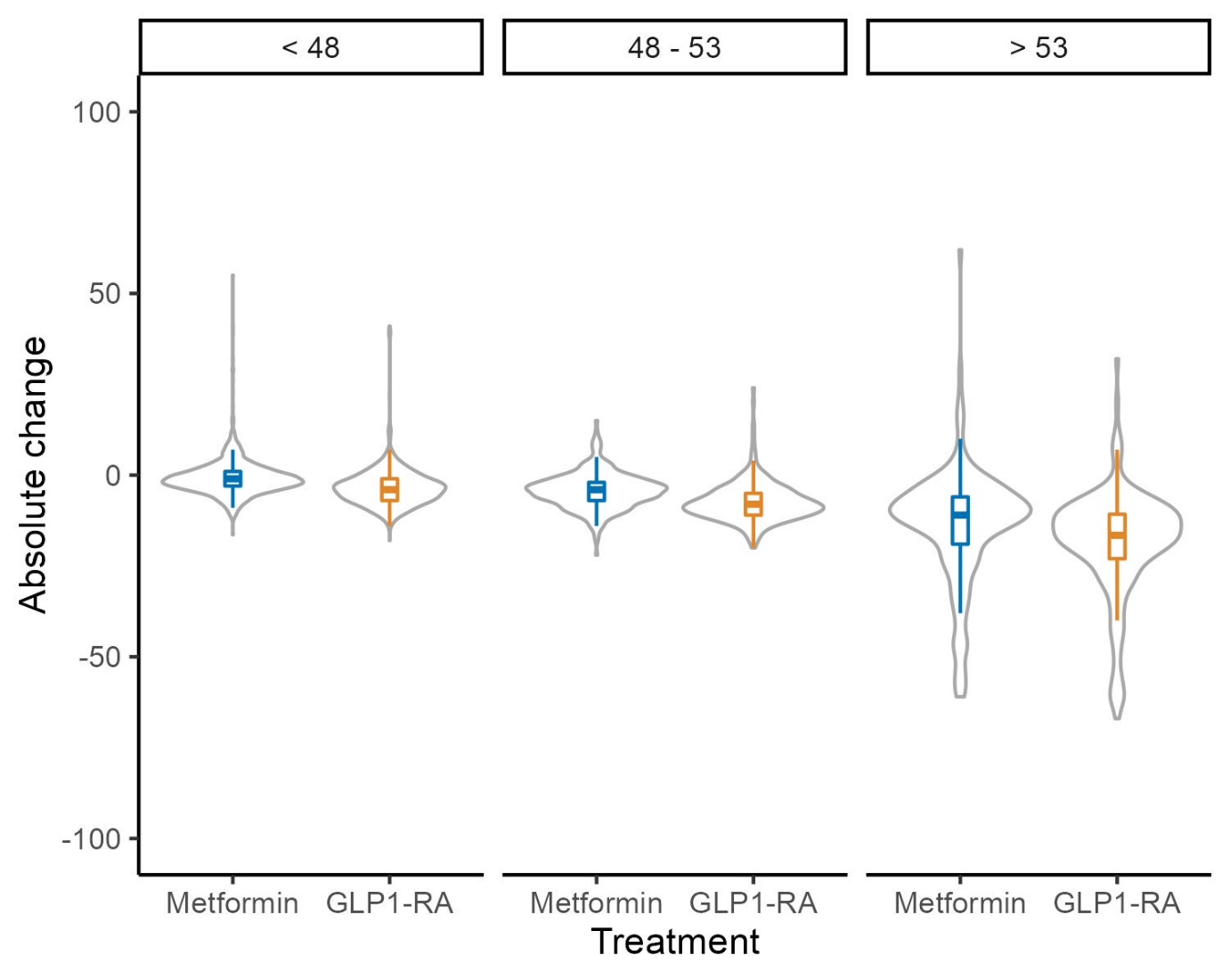


Figure S4: Difference between baseline [estimated glomerular filtration rate](https://www.kidneyfund.org/all-about-kidneys/tests/blood-test-egfr) (eGFR) (ml/min/1.73m2) and first available measurement during one-year follow-up, by baseline value of eGFR. Embedded box plot indicating the median and interquartile range. Metformin; n=915, GLP-1 RA; n=637


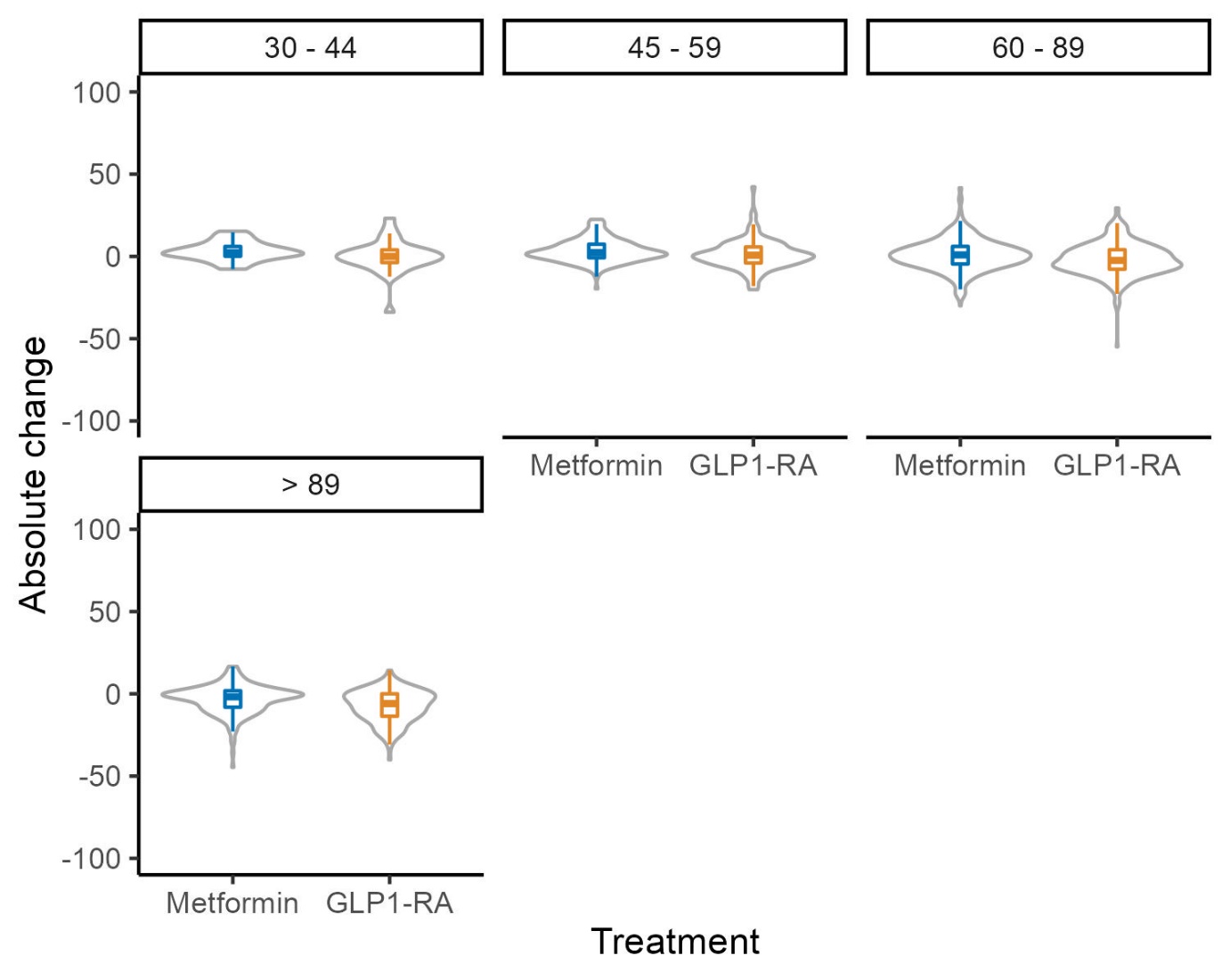


Figure S5: Difference between baseline LDL cholesterol (mmol/L) and first available measurement during one-year follow-up, by baseline value of LDL cholesterol. Embedded box plot indicating the median and interquartile range. Metformin; n=753, GLP-1 RA; n=550


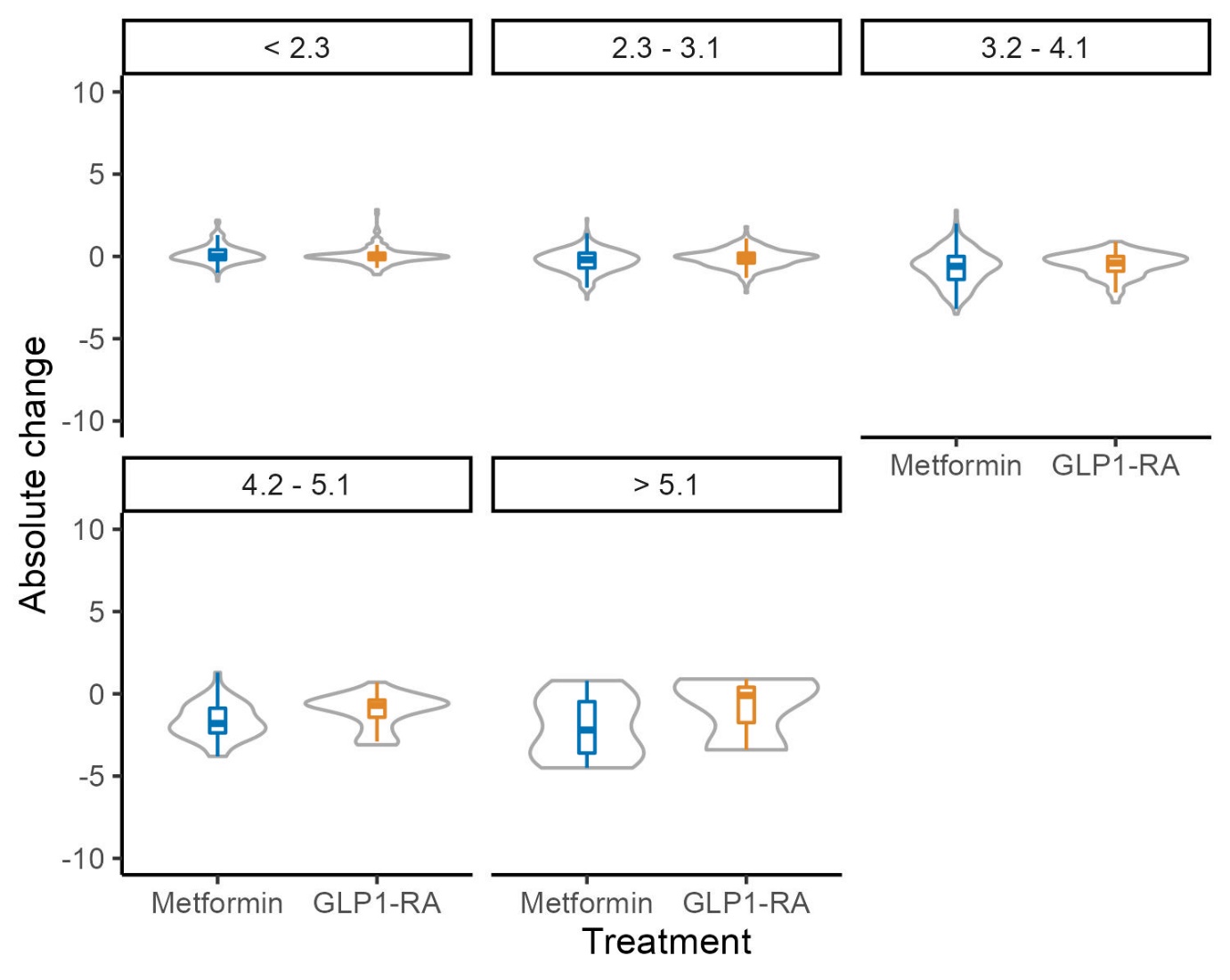


Figure S6: Difference between baseline triglycerides (mmol/L) and first available measurement during one-year follow-up, by baseline value of triglycerides. Embedded box plot indicating the median and interquartile range. Metformin; n=1269, GLP-1 RA; n=1102


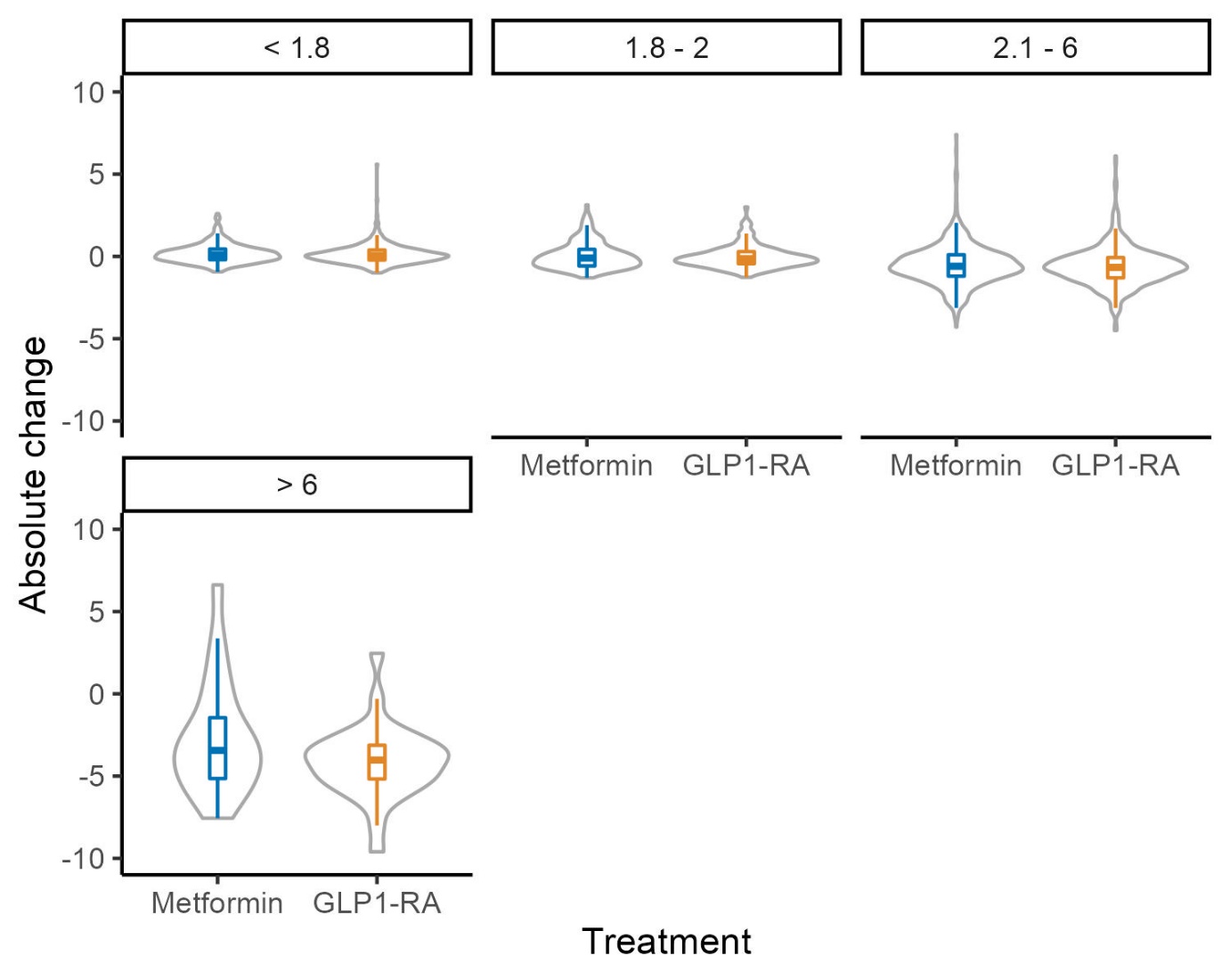


Figure S7: Average difference in effect on biomarker outcomes for first-line initiators of GLP-1 RA compared to metformin (reference), in subgroups of sex, age, previous cardiovascular disease (heart failure, stroke, ischemic heart disease, or peripheral vascular disease), and hypertension


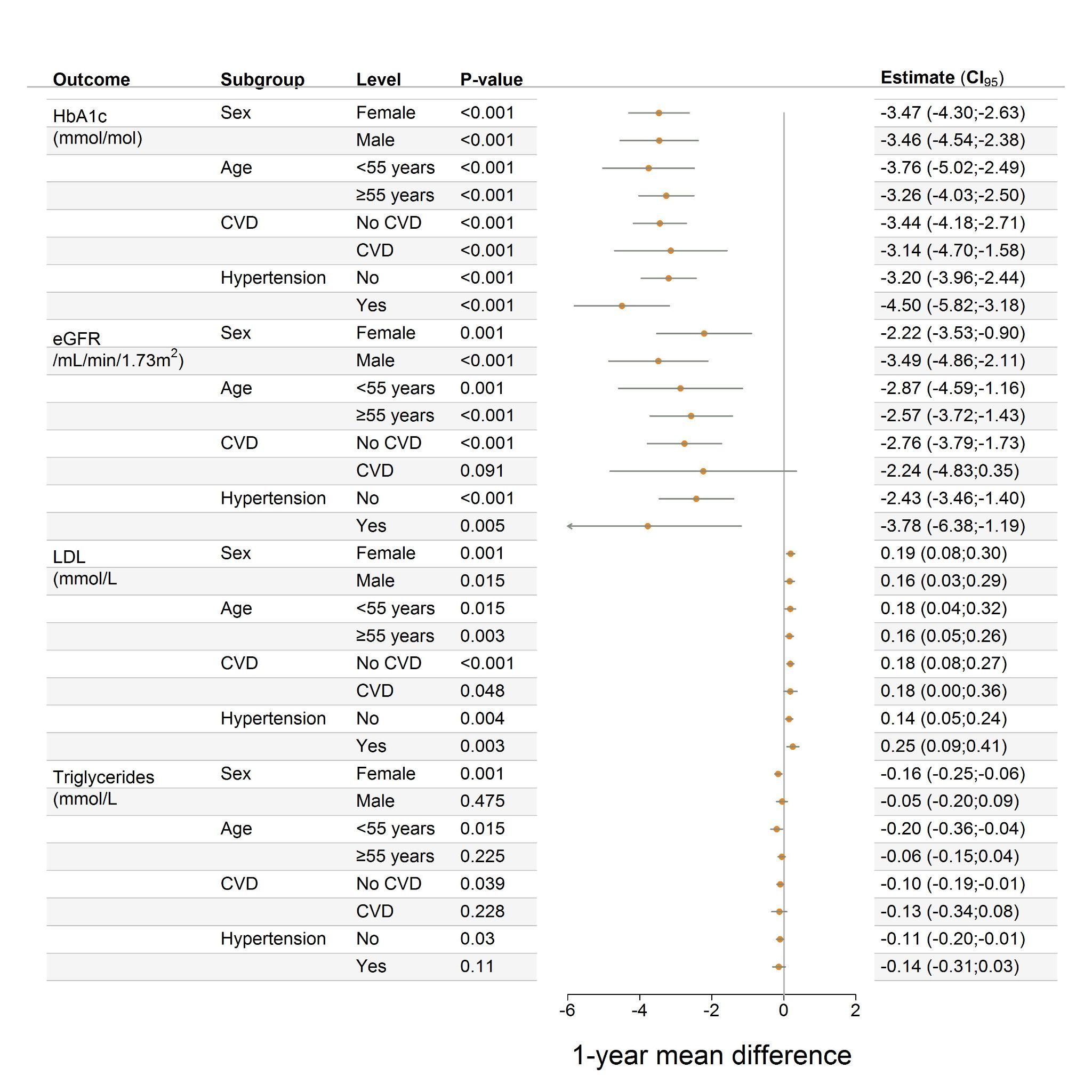


Figure S8: Probability of time to first biomarker measurement, A: HbA1c, B: eGFR, C: LDL cholesterol, D: triglycerides, with death as competing risk and emigration, end of follow-up (one-year) as censoring, by treatment


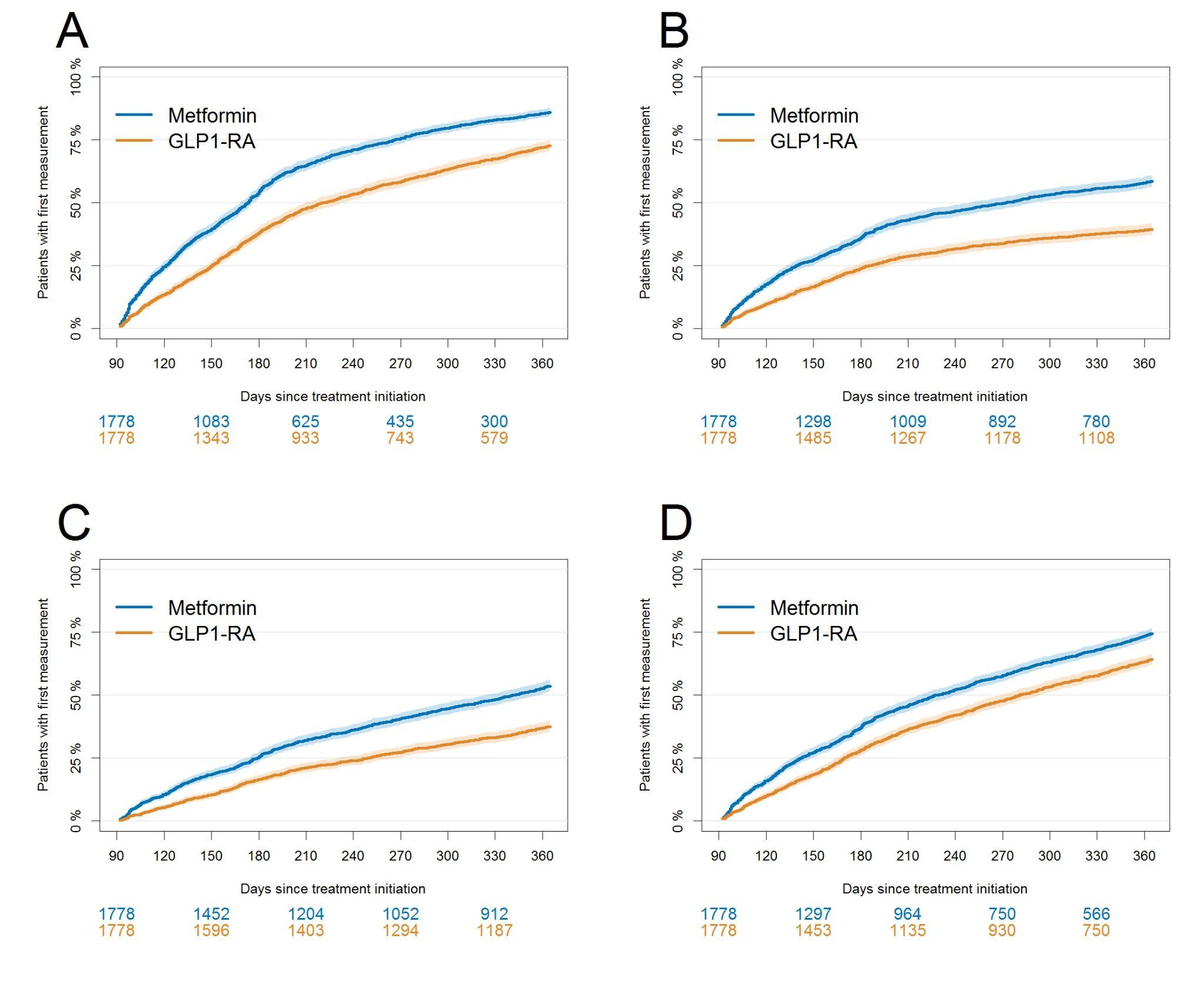

Supplement: Supplementary file 1 — Data S1. Supporting Information. [file JDB-16-e70000-s001.docx]
